# Human Microbiome: When a Friend Becomes an Enemy

**DOI:** 10.1007/s00005-015-0332-3

**Published:** 2015-02-15

**Authors:** Magdalena Muszer, Magdalena Noszczyńska, Katarzyna Kasperkiewicz, Mikael Skurnik

**Affiliations:** 1 Department of Microbiology, University of Silesia, Katowice, Poland; 2 Department of Bacteriology and Immunology, Haartman Institute, Research Programs Unit, Immunobiology, University of Helsinki, Helsinki, Finland; 3 University Central Hospital Laboratory Diagnostics, Helsinki, Finland

**Keywords:** Microbiome, Bacteria, Homeostasis, Health

## Abstract

The microorganisms that inhabit humans are very diverse on different body sites and tracts. Each specific niche contains a unique composition of the microorganisms that are important for a balanced human physiology. Microbial cells outnumber human cells by tenfold and they function as an invisible organ that is called the microbiome. Excessive use of antibiotics and unhealthy diets pose a serious danger to the composition of the microbiome. An imbalance in the microbial community may cause pathological conditions of the digestive system such as obesity, cancer and inflammatory bowel disease; of the skin such as atopic dermatitis, psoriasis and acne and of the cardiovascular system such as atherosclerosis. An unbalanced microbiome has also been associated with neurodevelopmental disorders such as autism and multiple sclerosis. While the microbiome has a strong impact on the development of the host immune system, it is suspected that it can also be the cause of certain autoimmune diseases, including diabetes or rheumatoid arthritis. Despite the enormous progress in the field, the interactions between the human body and its microbiome still remain largely unknown. A better characterization of the interactions may allow for a deeper understanding of human disease states and help to elucidate a possible association between the composition of the microbiome and certain pathologies. This review focuses on general findings that are related to the area and provides no detailed information about the case of study. The aim is to give some initial insight on the studies of the microbiome and its connection with human health.

## Introduction

The human body has generally been regarded as a self-sustaining organism that can regulate all of its life processes. However, over the past several years, researchers have shown that the human body resembles an ecosystem that consists of trillions of bacteria and other microorganisms. It is likely that the human ecosystem is the result of the evolutionary co-existence between the microbial community and the human body. Microorganisms inhabit almost every corner of the human body, including the gastrointestinal, respiratory, urogenital tracts and the skin. They are involved in important physiology functions in humans, such as digestion and the stimulation of the immune system (Ackerman 2012).

The composition of the human microbiome (microbiota) is highly personal and, therefore, it is challenging to clearly define “a healthy microbiome”. It was shown that the diversity in the composition of the microbiome among the body sites is greater than it is between individuals. This indicates that the human microbiome is highly variable ecosystem that possesses diverse microbiological parts (Proctor 2011; Ursell et al. 2012). However, it is possible to define “the core” of a healthy microbiome that occurs frequently within different body sites.

The human digestive system is a very complicated system that is composed of functionally distinct regions: the oral cavity, stomach, small intestine and colon. The human oral cavity is the perfect habitat for microorganisms due to the abundance of nutrients. The mouth is home to at least six billion microorganisms that belong to the *Firmicutes* (Gram positive; e.g., *Bacilli*, *Clostridia*), *Proteobacteria* (Gram negative, e.g., *Salmonella*, *Escherichia*, *Helicobacter* and *Yersinia*), *Bacteroidetes* (e.g., *Prevotella*, *Bacteroides*), *Actinobacteria* (Gram positive, e.g., *Actinomyces*, *Streptomyces*) and *Fusobacteria* (Gram negative, e.g., *Fusobacterium*) (Dave et al. 2012). Studies on gastric microflora have shown that it is composed mostly of *Actinobacteria*, but because of the acidic environment, *Helicobacter* (e.g., *H. pylori*) is also present (Bik et al. 2006). The small intestine microflora is qualitatively similar to the colon microbiome, but the latter contains more microorganisms. The proximal part of the small intestine contains relatively few bacteria, and mainly composed of Gram-positive *Lactobacillus* and *Enterococcus faecalis*. More microorganisms occur in the distal part, e.g., coliforms and *Bacteroides*. Nine major types of bacteria are found in the colon, although quantitatively *Firmicutes* and *Bacteroidetes* were dominant. At the genus level, anaerobic *Bacteroides* and anaerobic lactic acid bacteria, e.g., *Bifidobacterium bifidum*, prevailed (Dave et al. 2012). The microbiome of the gut performs extremely important functions for the normal development and functioning of the human body. These functions include the synthesis of vitamins, the decomposition of chemicals and nutrients, the support of fat metabolism, the outcompeting of pathogens, the promotion of angiogenesis and the maintenance of homeostasis and the development of the immune system (Holmes et al. 2011).

Analysis of the 16S rDNA sequences of the skin demonstrated that it is occupied by 19 phyla, but that the majority of the sequences was allocated to four phyla: *Actinobacteria* (51.8 %), *Firmicutes* (24.4 %), *Proteobacteria* (16.5 %) and *Bacteroidetes* (6.3 %). These dominant phyla were also present in the gut microbiome, but in different proportions. The most abundant were *Corynebacteria* (22.8 %—*Actinobacteria*), *Propionibacteria* (23.0 %—*Actinobacteria*) and *Staphylococci* (16.8 %—*Firmicutes*) (Grice et al. 2009; Grice and Segre 2011).

The human microbiota is altered over time due to the changing lifestyles of people. The excessive use of antibiotics and unhealthy diets pose a serious danger to the composition of the microbiome. At the same time, these factors destabilize the homeostasis of the microbiome as well as the homeostasis of the human body (Ursell et al. 2012). This microbiota has been extensively studied as part of many international research projects such as the Human Microbiome Project (HMP; www.hmpdacc.org), which was launched in October 2007 by the National Institutes of Health. The HMP brought together a huge number of experts and researchers to (1) characterize the communities of microorganisms that are found in the major ecological niches in humans, (2) assess the ecology of microbial metabolic and functional pathways, (3) understand the mechanisms that are responsible for the differences and similarities in the microbes people share and (4) determine their functional roles in health maintenance and disease development. These researches have led to over 350 papers (Gevers et al. 2012; Human Microbiome Project 2012a, b; Koren et al. 2013; www.ploscollections.org/hmp). Depending on the choices of parameters, the HMP estimates that the human microbiome contains between 3,500 and 35,000 Operational Taxonomic Units (OTUs). An OTU is a cluster of organisms that are grouped based on sequence similarity (Human Microbiome Project 2012a). In addition, the HMP discovered several novel taxa at the genus level. The abundance of these novel OTUs were <2 %, but they were present in significant number of volunteers. The novel taxa included the *Dorea*, *Oscillibacter* and *Desulfovibrio* genera, which correlated with disease states (colorectal adenoma, dietary shifts and opportunistic infections, respectively), the *Barnesiella* genus and a possible novel family within *Clostridiales* (Human Microbiome Project 2012a; Morgan et al. 2013; Wylie et al. 2012). Moreover, the HMP has supported the development of new technological tools and bioinformatics that allowed, e.g., whole-genome shotgun metagenomic data to be composed (Ravel et al. 2014). These data enable the universality of the concept of enterotypes in the human microbiota to be analyzed. It has been estimated that there are three distinct ecosystems—enterotypes in the human gut microbiome. These enterotypes vary in species, functional composition and enzyme balance. It has also been demonstrated *inter alia* that the enzymes which are associated with the biotin biosynthesis pathway are overrepresented in Enterotype 1, while those which are connected with the thiamine and heme biosynthesis pathways are dominant in Enterotype 2 and 3, respectively (Arumugam et al. 2011). Koren et al. (2013) use the term “enterotype” not only within microbial types in the gut, but also for different body sites. It was found that most samples fell into gradients that are based on bacterial taxonomic abundances. It was also determined that some body niches show a bimodal or multimodal distribution of the abundances of samples across the gradients (Koren et al. 2013).

Despite the huge progress in the field, the interactions between the human body and its microbiome still remain largely unknown (Ursell et al. 2012). Nevertheless, it is very important to highlight the larger role that the human microbiota plays in the development and maintenance of disease states.

## Digestive System

### Obesity

Traditionally, obesity is associated with energy disorders and an excessive intake of nutrients that is sometimes combined with a genetic predisposition. Recent studies have provided new information about the microbiome in the gut, especially its role in gaining an understanding of the pathogenesis of obesity. Observations have focused on the role of the gut microbiome in the development of obesity using rodent models (Harley and Karp 2012; Holmes et al. 2011).

It has been shown that the consumption of high-fat products reduces the total volume of the intestinal microbiome and induces the growth of Gram-negative bacteria (Holmes et al. 2011). According to this, there is a correlation between the composition of the microbiome and obesity. The intestinal microbiome differs between normal mice and obese mice, particularly in relation to the proportion of two bacterial groups—*Firmicutes* and *Bacteroidetes*. Obese mice exhibited a 50 % lower frequency of *Bacteroidetes* and an increased proportion of *Firmicutes*. Additionally, a significant increase in the number of genes that are associated with the use of energy from food was observed in the same population of obese mice. Consequently, scientists focused on initial studies of obese patients who were on a low-calorie diet. These analyses confirmed the results that had been obtained in the gut microbiome of mice. While an individual is on a diet, the frequency of *Bacteroidetes* increases, while the proportion of *Firmicutes* declines relative to the initial value. After losing weight for a year, the proportion of *Firmicutes* and *Bacteroidetes* in their intestinal microbiome was comparable with that found in slim individuals (Ley et al. 2005; Tlaskalová-Hogenová et al. 2011). Other studies showed that germ-free (GF) mice are not as likely to gain weight as slim ones—despite consuming the same amount of fat and carbohydrates. When they were colonized with the microbiome from obese mice, there was a tendency of faster fat deposition in comparison to the colonization with the microbiome from slim mice (Turnbaugh et al. 2008).

Since the correlation between the microbiome and obesity in rodent models has been established, scientists have focused on initial studies among humans. An analysis of 16S rRNA was performed on patients who were on a low-calorie diet. These analyses confirmed the results that had been obtained in the gut microbiome of mice—if obese patients lost weight over a period of a year, the proportion of *Firmicutes* and *Bacteroidetes* in their intestinal microbiome was comparable with that found in slim individuals (Harley and Karp 2012; Ley et al. 2006).

Studies have also shown that the gut microbiome controls an important gut-derived protein that is associated with host lipid metabolism, which is an angiopoietin-like protein 4 (Angptl4) that is also known as Fiaf. Angptl4 regulates the oxidation of fatty acid in both muscle and adipose tissue. When GF mice were colonized with the microbiome from obese rodents, the production of Angptl4 was suppressed in the intestine and more triglycerides were deposited in adipose tissue (which leads to a weight gain). Studies among humans have shown that a functional *angptl4* gene variant was more common in patients who had relatively low levels of triglycerides. Angptl4 can be an important regulator of lipid metabolism in humans (Bäckhed et al. 2007; Ley 2010).

Many studies include the correlation between the presence of *Helicobacter pylori* and obesity. The presence of *H. pylori* causes a postprandial decrease of ghrelin, a peptide hormone that is involved in the regulation of appetite. Gastric secretion of ghrelin significantly increased after the eradication of the bacterium and caused weight gain. Although the connection between *H. pylori* and obesity has been demonstrated in numerous studies, the role of ghrelin in the regulation of this process is still unclear. Further studies are needed to clarify the interaction between the factors that mediate weight gain and the eradication of the bacterium from the human body (Boltin and Niv 2012).

It was recently established that the endotoxin-producing *Enterobacter* induces obesity and insulin resistance in GF mice. During clinical studies, in a morbidly obese volunteer (weight 174.8 kg), who was suffering from serious metabolic deterioration, *Enterobacter* represented 35 % of his gut bacteria. After a diet, this amount decreased to less than 1.8 %. Further studies were performed on GF mice on a high-fat diet that were colonized with *Enterobacter* strain (*E. cloacae* B29) that had been isolated from the gut of an obese volunteer. Obesity and insulin resistance were observed in these mice. According to these studies on human-derived *Enterobacter* in GF mice, this bacterium may be involved in the development of obesity in humans (Fei and Zhao 2012).

### Gastrointestinal Cancers

According to the studies and experimental animal models, there is also a correlation between the composition of the gut microbiome and gastrointestinal cancers. It has been shown that the Western-style diet (a great deal of red meat and fat coupled with a low intake of vegetables) changes the intestinal microbiome—by increasing the activity of bacterial enzymes and the metabolism of bile acid. They can produce carcinogens or metabolize certain compounds into biologically active ones, which may play a role in carcinogenesis (Tlaskalová-Hogenová et al. 2011; Vannucci et al. 2009). Examples of these are the heterocyclic amines that are found in grilled meat, which are digested by bacteria in the colon and converted into electrophilic derivatives that can damage DNA and increase the risk of colon cancer. A high protein diet also provides the sulfur-reducing bacteria (such as *Desulfovibrio vulgaris*) with raw materials for the creation of harmful compounds—such as hydrogen sulfide. It can also damage DNA and increase the risk of colorectal cancer (Huycke and Gaskins 2004; Rooks and Garrett 2011). The highest production of carcinogens was associated with intestinal anaerobes and was reduced by supplementation with *Lactobacillus* (Chung et al. 1992).

Other effects of the Western diet also include changes in the bile composition—by increasing the proportion of secondary bile acids, which are the products of the transformation of primary ones by intestinal bacteria. One of these, deoxycholic acid, is associated with several models of carcinogenesis and the enzymatic activity of 7a-dehydroxylating bacteria (some *Clostridia*), which may be a target in the study of risk factors for gastrointestinal tumors (Holmes et al. 2011; Reddy et al. 1996).

A *H. pylori* infection has been recognized as a major risk factor for gastric cancer. Although more than 50 % of the world’s population is infected with this bacterium, less than 2 % of population develops cancer. Therefore, there are probably other risk factors (such as genetic, lifestyle, environmental or epigenetic aspects) that may play a role in the development of the disease (Conteduca et al. 2013). An infection of the gastric mucosa by *H. pylori* creates conditions that are favorable for the development of ulcers and lymphoma, which change the paths of mucin production, metaplasia and proliferation. These abnormalities are described in gastric and colorectal cancer (Babu et al. 2006; Tlaskalová-Hogenová et al. 2011). It was recently shown that variations in specific intestinal microbiome members affects an *H. pylori*-triggered inflammation. Antibiotic-treated mice were resistant to the inflammation and the alternations in the intestinal microbiome resulted in a decreased amount of Th1, which suggests a reduction in the Th1-promoting microbe or increased amounts of a Th1-inhibiting species. Furthermore, these mice had more *Clostridium* spp. (cluster IV and XIVa), which can prevent inflammation in the intestine by altering the recruitment of regulatory T cells to the gastric compartment. These indicate that manipulations of the gastric microbiome can set a new direction in the diagnosis and prevention of *H. pylori*-associated infections, e.g., gastric cancer (Rolig et al. 2013).

The composition of the intestinal microbiome is important for the appropriate homeostasis of the human body. Studies have shown that microorganisms can also affect the health of an organism in an indirect way. Several diseases of the digestive tract are connected with the immune system and its production of the relevant components. One of these disorders is inflammatory bowel disease (IBD).

### Inflammatory Bowel Disease

IBD is a chronic, relapsing inflammation of the gastrointestinal tract that results in a disruption of immune tolerance to the intestinal microflora and leads to mucosal damage in individuals who are genetically predisposed. Crohn’s disease (CD) and ulcerative colitis are usually included in IBD, although these two illnesses have a different pathogenesis and inflammatory profile (Fava and Danese 2011). The composition of the intestinal microflora and its activity in patients with IBD differ from the norm, mainly because of a lower incidence of dominant commensals (e.g., *Firmicutes*, *Bacteroides*, *Bifidobacterium*). A low number of *Firmicutes* leads to a decrease in the population of *Clostridium* of the IXa and IV groups, which are the main butyrate-producing bacteria. Butyrate affects the inhibition of the NF-κB (nuclear factor of kappa light polypeptide gene enhancer in B cells), thereby lowering the levels of pro-inflammatory cytokines. The NF-κB is a signaling module that plays a critical role in the immune system (e.g., regulates the expression of cytokines). Simultaneously, there is an increase in the occurrence of *Proteobacteria* and *Actinobacteria*, which are harmful sulfate-reducing bacteria as well as some *Escherichia coli* (Fava and Danese 2011; Frank et al. 2007).

The efficacy of the intestinal mucosa depends on an undamaged epithelium, secretion of antimicrobial peptides (e.g., defensins, IgA) and phagocytosis. The mucosal defense mechanisms are disturbed at all of these levels in IBD, which can lead to the progression of the disease. When membrane defensins and IgA decrease, there is an abnormal phagocytosis and hyperactivity of the immune response, which are considered to be the basis of the pathogenesis of IBD. The effect of commensal *Bacteroides fragilis* on IBD has also been investigated. The polysaccharide (PSA) molecule that is produced by this bacterium seems to suppress Th17 cells, which leads to a decrease in the pro-inflammatory response that causes colitis. Th17 cells produce interleukin (IL)-17, which is found at the elevated levels during an episode of the inflammation of the mucous membrane of patients (Mazmanian et al. 2008; Troy and Kasper 2010).

Some of the symptoms of CD are associated with polymorphisms in the NOD2/CARD15 gene (single nucleotide-binding oligomerisation caspase recruitment domain 15). The product of this gene, NOD2 protein, is a receptor that is present on the surface of host cells and it is involved in the regulation of the production of pro-inflammatory NF-κB-dependent factors. The activation of NOD2 by the peptidoglycan of the bacterial cell wall leads to the expression of α-defensins. Its expression was significantly inhibited within the small intestine in patients with CD (Binek 2012; Franczuk and Jagusztyn-Krynicka 2012; Kobayashi et al. 2005).

Experimental data have shown that single nucleotide polymorphisms in the NOD2 gene are not the only cause of the disease’s symptoms. *E. coli*, which has been detected in tissues of patients with CD, shows atypical peculiarities (both phenotypic and genotypic) (Baumgart et al. 2007). According to the pathogenic properties of the bacteria, it was called AIECE (adherent-invasive *E. coli*) and was found to be a new *E. coli* pathotype. This pathotype is able to invade inside the cells of the mucosa epithelium, where it changes the cell metabolism by disrupting the secretion of cytokines and interleukins. They may also be involved in causing inflammation (Boudeau et al. 1999). Not only are adherent-invasive strains of *E. coli* in the list of potential etiological factors of CD. Among others, these factors may be, e.g., the presence of *Listeria monocytogenes*, *H. pylori* and *Mycobacterium avium* subsp*. paratuberculosis*. The etiology of CD requires further studies (Fava and Danese 2011; Franczuk and Jagusztyn-Krynicka 2012).

Other studies have also suggested that IBD is associated with dysbiosis, which is characterized by changes in populations between *Firmicutes* and *Proteobacteria*. The question of whether this unbalance is a cause or a consequence of the intestinal inflammation requires further studies. It is very likely that dysbiosis, which is the lack of beneficial bacteria and a genetic predisposition, increases the permeability of the epithelium, impairs the immune response and causes a loss of tolerance to natural microflora. Additional approaches are needed including transcriptomics, proteomics, metabolomics and dietetics to determine the impact of changes in the human microbiome and the effects of specific mechanisms that result from the metabolic activity of microorganisms (Fava and Danese 2011; Morgan et al. 2012).

## Integumentary System

The integumentary system consists of the largest organ in the body, the skin and its associated structures, such as the hair and nails. The skin is colonized by a diverse collection of microorganisms including bacteria, fungi and viruses. The composition of the skin microbiome is complex, site-specific and depends on the location on the skin and its physiology (Grice and Segre 2011). To understand the bacterial influence on human health, scientists began to analyze the microbiome of the skin in several pathological conditions, such as atopic dermatitis (AD; also called eczema), psoriasis and acne (Kong 2011). All of these diseases appear to be directly connected with changes in the composition of the microbial community.

### Atopic Dermatitis

AD is a chronic, relapsing and intensely pruritic inflammatory skin disorder. It affects more than 15 % of children and 2 % of adults in the United States and 38 % and 10 % in Poland, respectively (http://www.naukawpolsce.pap.pl). The number of children suffering from this disease has been on the rise (almost tripled) in industrialized countries over the past 30 years, which suggests the influence of environmental factors (Kong et al. 2012).

Studies that are focused on the presence of *Staphylococcus aureus* on the skin of patients and healthy individuals have shown that in about 90 % of patients who suffer from AD, *S. aureus* affected both healthy and unhealthy areas of the body. By contrast, *S. aureus* was very rare on the skin of individuals who were not affected by the disease. Moreover, when a patient’s condition worsens, *S. aureus* often surpasses the entire community of microorganisms, thereby reducing the microbial diversity of the skin (Iwase et al. 2010; Kong et al. 2012). Metagenomics has shown that the proportion of *Staphylococcus* species that are present in the skin microbiome increased from 35 to 90 %, but surprisingly in addition to *S. aureus, S. epidermidis* was also involved. The latter can produce molecules that selectively inhibit *S. aureus*, which suggests an antagonistic relationship, even though both of these species may also interact mutualistically to strengthen their intestinal colonization (Iwase et al. 2010; Stecher et al. 2010). It is not exactly clear whether these *Staphylococcus* species promote each other’s growth or whether the growth of *S. epidermidis* is a response to the growing population of *S. aureus*. Understanding the implications of *S. aureus* on the skin microbiome can set a new course for the treatment of AD, which would be based on the manipulation of the composition of the skin’s microbiome and not only on the elimination of this bacteria (Chen and Tsao 2013; Kong et al. 2012).

### Psoriasis

There are some tested and proven methods for AD treatment including antibiotics or steroids, but there are no effective antimicrobial treatments for psoriasis (Trivedi 2012). Psoriasis is a common chronic inflammatory disease of the skin that is present in about 2 % of the world’s population (Schön and Boehncke 2005). The causes of this illness are poorly understood. It has been suggested that *S.* *aureus* and *Streptococcus pyogenes* infections might play a role in psoriasis, but treatment against *Streptococcus* was not effective and did not cure or relieve the disease state (Kong 2011; Weisenseel and Prinz 2005).

16S rDNA PCR for Archaea and bacteria was performed to examine the microbiome of normal and psoriatic skin. A greater variety of skin microflora was observed in ill patients, compared to healthy patients. The phylum *Firmicutes* was significantly overrepresented compared to the samples from uninvolved skin (both of the patients and healthy individuals). Actinobacteria were more numerous on the skin of healthy individuals and underrepresented in the psoriatic lesion samples, while the number of *Proteobacteria* was higher on the skin of the ill patients. A greater number of *Staphylococci* and a reduced number of *Propionibacterium* (*P. acnes* in particular) were also reported in the patients with psoriasis when compared to healthy individuals (Fahlén et al. 2012; Gao et al. 2008). Therefore, it has been suggested that the disease not only depends on fluctuations of the skin microbiome, but also appears to result from a combination of genetic and environmental factors (Gao et al. 2008; Kong 2011).

### Acne

Acne is a common skin disease that affects 85 % of teenagers (Webster 2002). It is well known that acne is associated with the occurrence of *Propionibacterium acnes* on the skin of patients who suffer from this ailment. Genotypic identification of microorganisms on skin (16S rRNA analysis) of healthy people and of patients with acne showed that healthy hair follicles contain only *P. acnes*, while the hair follicles in patients with acne are colonized by a mixture of *S. epidermidis*, *Corynebacterium spp.* and *P. acnes* (which predominated) (Bek-Thomsen et al. 2008). To better understand the strain diversity of *P. acnes* and their different roles in the disease, the genomes of 82 strains were compared. According to the results, acne was caused by certain strains of a species rather than the entire species, which is in line with the studies of other diseases (Kong and Segre 2012; Tomida et al. 2013).

It has recently been shown that skin that is inhabited by *P. acnes* can be divided into two populations—epidermal and follicular. To date, it has not been proven that epidermal *P. acnes* is an inflammatory trigger, but when it is present in hair follicles, it may contribute to inflammatory changes, e.g., acne. The conditions that are related to this process are as yet unknown (Alexeyev and Jahns 2012; Bojar and Holland 2004).

It is believed that microorganisms might be connected in the development of various skin disorders. Many aspects of the possible role of microbes in diseases of the skin and their cooperation with genetic and environmental changes are still unknown and further studies are warranted (Kong and Segre 2012).

Bacterial communities of the skin are also involved in immune homeostasis and inflammatory responses. It has been established that staphylococcal lipoteichoic acid (LTA) inhibits inflammation of the skin. LTA inhibits both the release of inflammatory cytokine from keratinocytes and the inflammation that is caused by an injury that is connected with Toll-like receptor 2 (Capone et al. 2011; Lai et al. 2009). It has been suggested that the immune pathways that are associated with the skin microbiome are linked to the development of allergies and asthma (Benn et al. 2002; Callard and Harper 2007; Capone et al. 2011).

## Immune System

The microbiome is essential in the activation of the host immune response and many autoimmune diseases result from a disturbance of the adaptive immune system. The frequency of autoimmune type 1 diabetes (T1D) and rheumatoid arthritis (RA) is increasing, which suggests that there has been a change in the environmental factors that regulate this system. An unbalanced diet, the widespread use of antibiotics and other social factors in developed countries may cause changes in the human microbiome and dysbiosis (Lee and Mazmanian 2010). The intestinal microbiome in healthy individuals is important and plays a unique role in the human body. This microbiome has been associated *inter alia* with metabolic functions (such as the digestion of various compounds and xenobiotics), in protective functions (i.e., the inhibition of an invasion of pathogens and the strengthening of the integrity of the epithelium) and in the modulation of the immune system (e.g., intestinal epithelial homeostasis) (Fava and Danese 2011).

Studies have shown the critical role that the immune system receptor (MyD88) plays in the recognition of microbes that occurs in immune homeostasis. MyD88-dependent bacterial signals induce the repair of a damaged intestinal epithelium (Pull et al. 2005) and promote the induction of epithelial antimicrobial proteins such as RegIIIγ. The expression of RegIIIγ may be triggered by lipopolysaccharide or flagellin (Brandl et al. 2007; Hooper et al. 2012; Kinnebrew et al. 2010).

The commensals that are present in the human body may induce the differentiation of CD4^+^ T cells into the four main types: Th1, Th2, Th17 and Treg (regulatory T cells). Each type has a different role in the immune system and secretes characteristic cytokines (e.g., interleukins). Th1 cells are involved in the elimination of intracellular pathogens and Th2 cells control parasitic contagion. Th17 cells play an important role in the control of infections, while Tregs regulate the immune response (Wu and Wu 2012).

The human microbiota modulates the proper balance between these four cell types. It has been established that segmented filamentous bacteria (SFB) play a role in the induction of Th17 and Th1 cells (Ivanov et al. 2009). Other bacteria (e.g., some Clostridia strains) increase the abundance of Treg cells and induce the expression of the inducible T cell co-stimulator and IL-10, which are important anti-inflammatory molecules (Atarashi et al. 2013).

SFBs induce many intestinal immune responses that support Th17 cell differentiation, such as the production of cytokines and chemokines, antimicrobial peptides and serum amyloid A. The colonization of animals by SFBs protects them from a *Citrobacter rodentium* infection that causes inflammation similar to enteropathogenic *E. coli* in humans (Lee and Mazmanian 2010; Snel et al. 1998).

Moreover, *Bacteroides fragilis* has a beneficial effect on the balance of the immune system. *B. fragilis* has become a model for the study of the correlation between symbiotic bacteria and the immune system. Bacterial PSA, which are produced by *B. fragilis*, stimulate the development of Treg cells and the production of increased amounts of IL-10. PSA is able to prevent and treat experimental autoimmune encephalomyelitis (EAE). This treatment results in an inhibition of pro-inflammatory cells and in an increase in the Treg numbers in the central nervous system (CNS). This suggests that the presence of *B. fragilis* is sufficient to determine the proper balance of Th1/Th2. Dysregulation of Th1 or Th17 activity may lead to autoimmune diseases, while overactivity of Th2 may be one of the causes of asthma and allergies (Ackerman 2012; Lee and Mazmanian 2010; Mazmanian et al. 2005; Troy and Kasper 2010).

### Type 1 Diabetes

An imbalance between *Firmicutes* and *Bacteroidetes* and a low diversity in the gut microbiome may cause T1D, which is also called insulin-dependent diabetes. It is a disease that results from the T cell-mediated slow destruction of insulin producing islet β cells in the pancreas. *Firmicutes* (more specifically *Lactobacillus*, which is present in the human body) induces Treg in the large intestine, as well as other organs, and supports the immune system homeostasis. Decreased amounts of Treg cells have been reported in patients with T1D, which suggests the participation of the intestinal microbiome in the development of the disease (Livingston et al. 2010; Romano-Keeler et al. 2012; Wu and Wu 2012). Furthermore, it has been proposed that *Lactobacillus johnsonii* may delay the progression of T1D by stimulating the development of Th17 (Boerner and Sarvetnick 2011; Vaarala 2011).

Experimental evidence has shown that non-obese diabetic (NOD) mice that lack the innate microbial-recognition immune system receptor, MyD88, are resistant to T1D. The protective effect of MyD88 deficiency requires the presence of the gut microbiome, since mice that were lacking MyD88 NOD readily developed diabetes in a GF facility. These results indicate that the protective effect of MyD88 deficiency is due to the induction of MyD88-independent signaling due to the expansion of beneficial bacteria (Wen et al. 2008; Wu and Wu 2012).

### Rheumatoid Arthritis

RA is an autoimmune disease that causes the chronic inflammation of the joints. Patients with this disorder display an abnormal circulation of Treg cells and an increased number of Th17 and IL-17 in the plasma and synovial fluid of the knee (Hot and Miossec 2011; Scher and Abramson 2011). On the other hand, infections of enteric pathogens such as *Salmonella*, *Yersinia* and *Shigella* can trigger autoimmune reactions in the joints and reactive arthritis as sequelae (Toivanen 2003). Many studies have tried to pinpoint the arthritogenic molecules of the bacteria; urease subunits of *Yersinia* have been suggested as an arthritogenic factor in a rat model (Gripenberg-Lerche et al. 2000). In addition, patients with RA had higher levels of antibodies against certain species of intestinal bacteria (e.g., *Proteus*) and *Klebsiella*, which suggests that there is a link between this bacteria and RA (Ebringer et al. 2010; Rashid and Ebringer 2007). Moreover, some antibiotics (e.g., sulfasalazine and minocycline) are reported to have a therapeutic effect for some patients, which may be related to the antibacterial activity of these molecules (Wu and Wu 2012).

The intestinal microbiome dysbiosis in susceptible individuals can potentially lead to a pro-inflammatory response that damages tissue (e.g., connective tissue of the joints) (Scher and Abramson 2011).

## Cardiovascular System

### Atherosclerosis

Atherosclerosis is a progressive process that causes focal thickening of muscular and large elastic arteries. Studies have shown a connection between periodontitis, *Chlamydia pneumoniae* and *H. pylori* infections and atherosclerosis (Desvarieux et al. 2005). Because of those relationships, attempts have been made to treat atherosclerosis with antibiotics. The results have not led to any consensus regarding the effect of antibiotics in preventing or reducing atherosclerosis. Studies using anti-chlamydial antibiotics have also not demonstrated any positive effects in patients with arterial disease (Jaff et al. 2009; Muhlestein 2003). Bacteria from the oral cavity may affect atherosclerosis by promoting low-grade inflammation. Almost all of the periodontal bacteria were detected in the atheromatous plaque samples that had been obtained from periodontitis patients. Their DNA constituted 47.3 % of the total bacterial DNA in the samples. *Prevotella intermedia*, *Porphyromonas gingivalis* and *Actinobacillus actinomycetemcomitans* were frequently identified in the plaques from patients with periodontitis, while *P. gingivalis* was the only targeted microorganism that was observed in these plaques from healthy subjects. According to these studies, the oral periodontopathic bacteria that are present in atherosclerotic tissue samples may contribute to the development of vascular diseases (Gaetti-Jardim et al. 2009).

Furthermore, researchers have shown that the microbiome may be important for the initiation, but not for the progression, of atherosclerosis (Caesar et al. 2010). Experimental studies using apolipoprotein E-deficient mice (ApoE^−/−^) attempted to confirm this. It has been shown that conventionally reared ApoE^−/−^ mice that had been fed a low-fat diet do not tend to develop atherosclerotic plaques, in contrast to the same type of mice that had been reared in GF conditions (Caesar et al. 2010). Differences in atherosclerotic plaques are not as apparent when mice are fed a high-fat diet that is supplemented with cholesterol (Stepankova et al. 2010). It was also demonstrated that the GF ApoE^−/−^ mice had slightly reduced atherosclerosis after 22 weeks of high-fat feeding (Wright et al. 2000). These results document that there is a connection between the microbiome and the development of atherosclerosis (Tlaskalová-Hogenová et al. 2011).

## Nervous System

Several neuropathological diseases are thought to be associated with the gut microbiome because of the interactions of the CNS and the gut (gut-brain axis). Neural, immunological and endocrinological mechanisms are involved in this communication. The enteric nervous system directly controls the functions of the gastrointestinal tract (Collins and Bercik 2009; Tlaskalová-Hogenová et al. 2011).

The use of GF mice has enabled the impact of the gastrointestinal microbiome and probiotics on behavior and behavioral abnormalities, including anxiety to be studied (Cryan and O’Mahony 2011). Studies suggest that the colonization of the gut microbiome has become integrated into the programming of brain development, thus affecting motor control and anxiety-like behavior. GF mice had increased motor activity and reduced anxiety, compared with specific pathogen-free mice with a normal gut microbiome. This is associated with changes in expression of the genes that are involved in the second messenger pathways and synaptic long-term potentiation in specific brain regions (Diaz-Heijtz et al. 2011).

There are several studies that have linked the composition of the microbiome to psychiatric disorders, such as autism spectrum disorders (which include autism) and multiple sclerosis (MS). This association also appears to be related to the metabolites of dietary components, in which the microbiome plays a major role (Gonzalez et al. 2011).

### Autism

Autism spectrum disorders (ASD) is a group of complex neurodevelopmental dysfunctions that are characterized by impairments in social interaction and communication, as well as repetitive behaviors. ASD include several disorders, one of which is autism. Autism is a neurodevelopmental ailment of a complex origin that is defined by social, cognitive and behavioral dysfunctions. In recent years, the incidence rate has begun to rise, which points to the environment as a contributing factor of autism. Environmental factors include exposure to certain chemicals, drugs, stress, infections (also maternal) and dietary agents (Dietert et al. 2011; Holmes et al. 2011; Louis 2012).

A prominent subset of ASD patients suffers from gastrointestinal (GI) symptoms for unknown reasons, which points to the role of gut microbiome in this disorder (Benach et al. 2012). The evidence of the microbial influence on ASD is supported by the fact that interventions using antibiotics and probiotics have positive effects on behavior and the neuropsychological symptoms (Critchfield et al. 2011). Such studies provided the evidence that *Sutterella* species are present in the ileal mucosal biopsy specimens from autistic patients, but not from healthy children with GI symptoms. This suggests that *Sutterella* may play a role in this disorder (Williams et al. 2012). Furthermore, a greater abundance of *Ruminococcus torques* was observed in the fecal samples of children with ASD and GI symptoms (Wang et al. 2013).

Some reports have indicated that fecal microbial profiles of autistic children are characterized by tenfold higher numbers of *Clostridium* spp. compared with healthy subjects (Finegold et al. 2002). Some *Clostridium* species (such as *C. histolyticum*), which are known to produce neurotoxins, occurred more frequently in the fecal samples from ASD children when compared to unrelated healthy individuals (Gonzalez et al. 2011; Parracho et al. 2005; Sekirov et al. 2010). In addition, the metabolism of *Clostridium* species may play a role in pathogenesis of autism. Treatment of regressive-onset autistic children with the antibiotic vancomycin resulted in improved behavior and communication skills. However, these gains were temporary and only lasted while the children were undergoing treatment. These studies suggest that autism may be connected with intestinal bacterial species that are sensitive to vancomycin (Critchfield et al. 2011; Sandler et al. 2000).

Moreover, multiple dysbiosis in fecal microbiome was also observed. At the phylum level, the proportion of *Bacteroidetes* increased, while the level of *Firmicutes* was lower (Finegold et al. 2010). *Desulfovibrio* was also found in significantly higher numbers in severely autistic children than in the controls, in contrast to some *Bifidobacterium* species, which decreased (Adams et al. 2011; Finegold 2011). Autism is also associated with metabolic alterations and microbial cometabolism. These include higher urinary levels of hippurate and phenylacetylglutamine, perturbations in sulfur, amino acid metabolism and the tryptophan/nicotinic acid metabolic pathway (Iebba et al. 2011; Yap et al. 2010).

A recent study showed the absence of *B. fragilis* in mice with the autism-like disorders compared to the wild type. When these mice were colonized with *B. fragilis* through food, both behavioral problems and gastrointestinal difficulties diminished. This finding can be useful not only in autism, but also in other neurodevelopmental disorders (Hsiao et al. 2013).

The fecal microbiome and metabolome of children with autism were established by pyrosequencing of the 16S rDNA and 16S rRNA. The highest microbial diversity was found in AD children. These results confirmed most of the earlier findings, including the amounts of *Bacteroidetes*, *Firmicutes*, *Saturella* and *Clostridium* in the microbiome of autistic children compared to healthy children. Furthermore, the levels of other Clostridia-related genera (*Caloramator* and *Sarcina*), Enterobacteriaceae, as well as *Alistipes* and *Akkermansia* species were higher in AD children compared to healthy ones. Conversely, Eubacteriaceae (except for *Eubacterium siraeum*) and *Bifidobacterium* species were found at lower level (De Angelis et al. 2013).

### Multiple Sclerosis

Experimental autoimmune encephalomyelitis (EAE) is a mouse model of multiple sclerosis (MS) in which an autoimmune response causes demyelination in the central nervous system (CNS). The pathological mechanism of EAE may differ significantly from human MS, but it can still give valuable information about the role of the microbiome in these diseases (Wu and Wu 2012). Bacterial involvement in the pathogenesis of MS was suggested because of the presence of bacterial peptidoglycan within the antigen presenting cells in the brains of patients. The earlier mentioned PSA, which is produced by *B. fragilis*, is able to prevent EAE by inhibiting pro-inflammatory cells, while increasing the amount of regulatory T cells in the CNS. Modification of the commensal bacteria by antibiotics modulates the peripheral immune tolerance that can protect against EAE and significantly weaken the disease. The protection is associated with a reduction of pro-inflammatory cytokines and an increase in IL-10 and IL-13 levels (Ochoa-Repáraz et al. 2009; Tlaskalová-Hogenová et al. 2011).

The lower production of pro-inflammatory cytokines, such as IL-17, was observed in GF mice. These rodents were colonized with SFBs, which are known to induce Th17 cells in the gut. Increased Th17 cell responses and the occurrence of EAE were reported after the colonization. These studies suggest that the microbiome is composed of organisms that can direct both pro- and anti-inflammatory immune responses in the CNS (Lee et al. 2011; Wu and Wu 2012).

There is evidence that *Clostridium perfringens* may be involved in inflammations (Rumah et al. 2013). Some *C. perfringens* species (e.g., type B and D) are known to produce epsilon toxin, which appears to be an MS trigger in individuals with a genetic predisposition. According to some studies, this toxin causes blood brain barrier permeability, kills oligodendrocytes and also targets the retinal vascular and meningeal cells that are involved in MS inflammation (Dorca-Arévalo et al. 2008, 2012; Rumah et al. 2013).

## Conclusion

The human microbiome is a remarkably variable ecosystem that has various microbiological niches whose diversity within body sites is greater than that between individuals. The role of the microbiome in human health has been studied by many international teams of scientists. The Human Microbiome Project is an interdisciplinary and global project, the main goal of which is to comprehend the microbial components and their influence on homeostasis and a predisposition to disease. The symbiosis between the human host and the microbiome maintains specific physiological responses. Shifts in the latter can impair many of those, so that they result in a variety of chronic, localized, sometimes acute and systemic human diseases, e.g., IBD, cancer, obesity and autism. The growing awareness of the importance of the microbiome in health and disease and a more comprehensive analysis of it may pave the way to a more complete knowledge of human physiology and to treating the human body as a “superorganism”, a complex ecosystem in which each part interacts and communicates with the others.
